# Hierarchical medical system and local medical performance: A quasi-natural experiment evaluation in Shanghai, China

**DOI:** 10.3389/fpubh.2022.904384

**Published:** 2022-10-17

**Authors:** Chen Liang, Yihang Zhao, Chenglong Yu, Peng Sang, Long Yang

**Affiliations:** ^1^School of International and Public Affairs, Shanghai Jiao Tong University, Shanghai, China; ^2^Faculty of Public Health and Policy, London School of Hygiene and Tropical Medicine, London, United Kingdom; ^3^School of Computer Science, Nanjing University of Posts and Telecommunications, Nanjing, China

**Keywords:** hierarchical medical system, policy effects evaluation, dynamic effects analysis, influencing mechanisms analysis, causal inference

## Abstract

**Background:**

In order to maintain high standards of healthcare, it is necessary for medical departments to provide high-quality and affordable medical services to local residents. This has been widely accepted in developed countries, while the medical treatment systems in developing countries remain to be improved. This research is based on a pilot of a hierarchical medical system in Shanghai, China, to evaluate the effects on policy of medical reform in developing countries.

**Methods and results:**

By means of the difference-in-differences (DID) method, the causal relationship between medical care services' improvement and hierarchical medical systems' implementation could be identified. This project also explores the differential effects of policy intervention and confirms that the pilot showed a significant improvement in medical performance in central districts while the result remains uncertain in terms of suburban districts. Furthermore, the dynamic effect of a hierarchical medical system has also been identified with the event study method, while the policy pilot only had short-term effects on local medical resources' improvement. In order to ascertain the function mechanisms of hierarchical medical systems and explain why the policy pilot only had short-term effects, this project also conducts influencing mechanism analysis with the triple-differences method (also known as difference-in-difference-in-differences or DDD method). According to the empirical results, there is no direct evidence indicating the hierarchical medical system could bring obvious benefits from the perspectives of patients and medical institutions.

**Conclusions:**

For better implementation of hierarchical medical systems in the future, long-term supervision mechanisms should be given more attention in the enforcement process of hierarchical medical systems. At the same time, more safeguarding measures should be implemented, such as supervising the payment systems of the medical institution and conducting performance evaluation.

## Introduction

Striving for accessible, high-quality, and affordable medical services has become a consensus for departments of health around the world (1). However, because of the enormous population and imbalanced development in China, especially considering the aging population (2), per capita medical resources are finite, and it is challenging for the Chinese government to make and implement the correct medical policies to cover everyone. At the same time, the economic gap between the western, central, and eastern parts of China is significant (3); the development difference between urban and rural areas is obvious, which directly or indirectly leads to a great imbalance in the distribution of medical resources in different regions (4, 5). To solve the current medical problems, the Chinese government continuously increases financial expenditure in the medical field, which does not effectively resolve regional differences and conflicts; financial expenditure occurs as necessary to strengthen policy arrangements at the institutional level to achieve a balanced allocation of medical resources–in a fundamental sense, to promote the fair distribution of medical resources (6). Hence, a forward-looking plan was carried out from the top down, that is, the hierarchical medical system in China.

**Figure 1:**
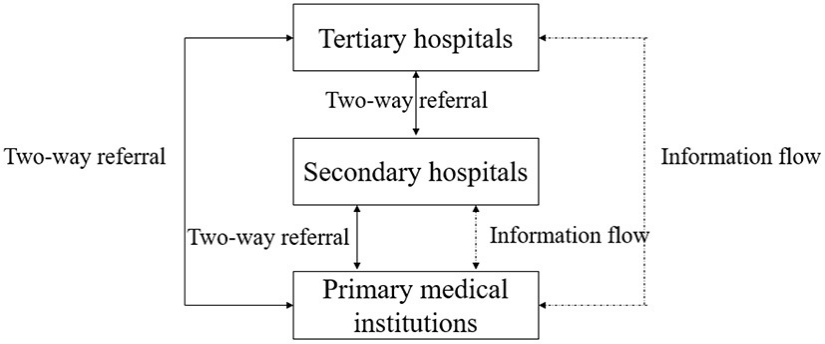
The theoretical model of a hierarchical medical system.

A hierarchical medical system is an ideal model and has gradually become an essential medical system in most developed countries (7, 8), guaranteeing basic health care (9). However, it is still in its infancy in China. Generally speaking, the hierarchical medical system requires that patients with minor illnesses go to primary medical institutions, while patients with serious illnesses must go to specialized medical institutions for diagnosis and treatment, and these patients should go to primary medical institutions for postoperative care when they are well again (10). According to the theoretical model of the hierarchical medical system in Figure 1, this model could greatly relieve the stress on large hospitals where a large number of patients attend, and could improve the efficiency of medical resource allocation.

There are three levels of medical departments in China, and the tertiary hospital is the highest level of hospital, which contains the most complete medical resources. As shown in Figure 1, when using the hierarchical medical system, all three levels of medical institutions have two-way referral with each other. Since the primary medical institution is the first place patients visit, it shares patients' information, including medical records, medical examination files, medication records, and other private information, with the higher ranking hospitals. This ensures continuous medical treatment for patients when they visit different medical institutions and saves time. In sum, when operating effectively, the hierarchical medical system could greatly improve the allocation of medical services and achieve equalization of public medical resources. In terms of patients, it could greatly cut down their medical expenditure.

Shanghai is one of China's biggest cities and has the most developed economic level. In terms of medical resources, there are 66 tertiary hospitals, 101,000 medical practitioners, and 118,000 medical beds in Shanghai.^1^ In China, the amount of medical facilities in Shanghai is second only to Beijing. However, the tertiary hospitals are usually overcrowded all year round. This could be attributed to the following reasons. Firstly, according to the “Investigation report on medical treatment of Shanghai residents in 2017”, 39.1% of respondents chose to directly go to a tertiary hospital when they felt sudden discomfort, while only 14.1% of the respondents chose to visit primary medical institutions; patients prefer visiting higher ranking medical institutions even if their symptoms are mild. Secondly, Shanghai has a large aging population. By the end of 2014, there were 4.19 million people aged over 60 in Shanghai, accounting for 28.8% of the registered population.^2^ Nationwide, 15.5% of the population are over 60, a percentage which is approximately half than that of Shanghai. This situation in Shanghai has contributed to great pressure on tertiary hospitals to admit patients, and it has been observed that supply exceeds demand of medical resources in primary medical institutions.

**Figure 2:**
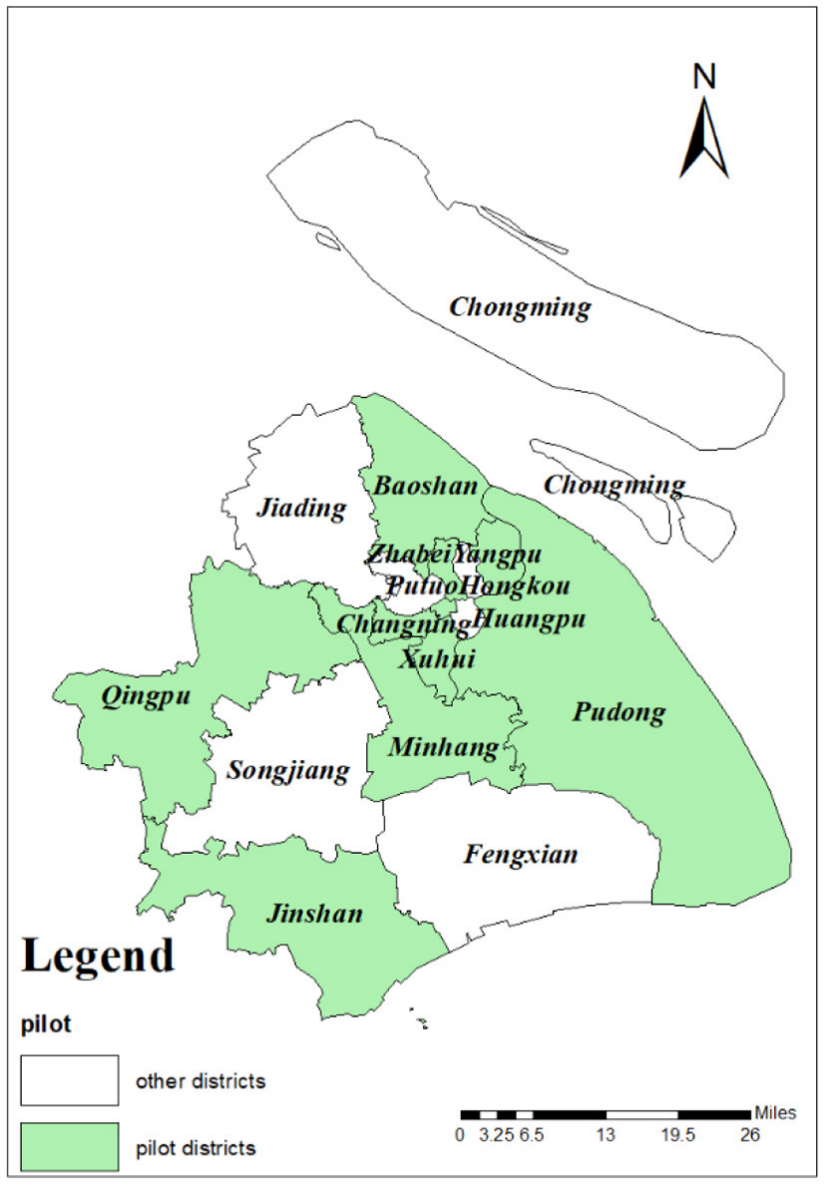
The distribution of pilot districts in Shanghai, China.

In order to relieve the medical pressure on tertiary hospitals and implement a rational allocation of medical services, Shanghai initiated implementation of the hierarchical medical system in 2011. As a pilot for nationwide policy promotion, the implementation experience provided objective indicators. The pilot areas were Changning, Xuhui, Pudong, Jingan, Zhabei (merged to Jingan district in 2015), Baoshan, Yangpu, Minhang, Qingpu, and Jinshan. As shown in Figure 2, pilot districts are indicated in green while districts with no pilot programme are shown in white. These 10 districts were randomly distributed across Shanghai, satisfying the pre-conditions of the DID method. In terms of its core operation mode, a network of family doctors was designed to cover all registered households. Referring to Figure 1, family doctors integrate basic and advanced medical resources, and provide guidance for patients to choose a related medical institution for their first visit. Family doctors could not only become “gatekeepers” of residents' health but also promote the rational allocation of medical resources and save patients' medical expenditure.

## Literature review

In essence, the medical hierarchical system is a policy arrangement that promotes the rational division of labor and cooperation among various medical institutions. The fairness and accessibility of medical resource allocation promote the separation of primary health care and specialized medical services in medical institutions at all levels. Specifically, basic medical care is provided by primary medical institutions, and specialized medical care is provided by higher-level hospitals. In the meantime, medical institutions cooperate in the fields of personnel training, scientific research, health education, and medical technology, which could promote the provision of continuous services for patients (11). However, it appears that huge differences in the operation processes of hierarchical medical mechanisms exist between developed and developing countries.

First, in developed countries, doctors of basic health care needs must be qualified as general practitioners. Developed countries focus on the training of general practitioners. Training systems in the USA and UK are very rigorous, and medical students require 11 years to complete their studies.^3^ In Germany, a medical student requires 10 years to complete their studies.^4^ Second, there are many regulations and laws in developed countries to ensure the efficiency of medical resource allocation. For example, in Italy, the amount of equipment, beds, and medical staff in a hospital are allocated according to a medical plan (12). Third, the specific function of each medical institution is clear, and each institution performs its duties efficiently. Compared to medical institutions in China, specialized hospitals in developed countries do not have outpatient departments; they only treat patients referred by general practitioners (13).

Compared to developed countries, most developing countries do not have adequate financial resources to afford high levels of medical expenditure (14), and in most cases, the hierarchical medical system pilot failed to improve the efficiency of medical institutions' operation (15). Though the medical system in China has undergone several reforms, it still has many shortcomings. For example, the focus of medical and health care has changed from prevention to treatment, and strict hierarchical diagnosis has been replaced by a more relaxed “hospital-centrism” medical treatment mode (16). Medical institutions have become more focused on making profits, contributing to a mismatch between medical resources and increases in medical expenditure (17). In 2009, the medical department in China issued “Opinions of the CPC Central Committee and the State Council on deepening the reform of the medical and health system”, which followed the hierarchical medical mode in developed countries and suggested implementing a clear division of labor, complementary functions, close cooperation, and efficient operation. This was in order to form an appropriate medical procedure of primary diagnosis, two-way referral, linkage between different ranks of medical institutions, and division of emergency and non-urgent treatment.^5^ Unfortunately, many proposed reform measures have not been actioned as yet (18). Without clear results from the policy changes made in the pilot, it is difficult to implement a hierarchical medical system on a large scale.

Scholars have carried out considerable research on Chinese hierarchical medical measures and concluded that there are many operational difficulties with the current situation in China. Firstly, the primary diagnosis system means patients being treated first at a primary institution, which may cause problems because patients do not trust primary medical institutions in China (19), and patients and medical insurance funds are still siphoned by higher rank hospitals (20). Secondly, in terms of the two-way referral system, in reality, the number of upward referrals (to secondary and tertiary hospitals) was significantly higher than that of downward referral (to primary medical institutions), which will meanwhile increase the burden on higher ranking hospitals (21). Some research has also analyzed approaches from the perspectives of government intervention and market regulation (22), which represent two areas that could have an effect on the hierarchical medical system. It is still uncertain which path to take –whether administrative measures should be taken to solve medical resource allocation or whether market-oriented health needs should be complied with which accord with “patients-centrism” (23).

According to the relevant research as mentioned above, since the recent rise of hierarchical treatment services, developed countries have typically adopted different integration models and strategies in medical services, and have achieved satisfactory results. However, the effect of the policy remains uncertain in developing countries. Shanghai was one of the first cities in China to pilot the hierarchical treatment system. Referring to the statistical data from the Shanghai Statistical Yearbook (2005–2016), regardless of medical resources and what treatment a patient had, an increasing trend in using the hierarchical treatment system was reported, which indicated that the pilot in Shanghai had achieved great progress. This research intends to fill the gap to evaluate the effects of the hierarchical treatment pilot in developing countries, with cautious empirical analysis of the causal inference method.

## Theoretical framework and research hypotheses

It is generally believed that the hierarchical medical system encouraged treatment in primary institutions and two-way referral within three-level medical hierarchies in China. The policy pilot required all of the medical institutions to share patients' information with the other hospitals in the hierarchy, including medical records, medical examination files, medication records, and other private information. This ensured the sustainability and stability of patients' medical treatments, thereby improving overall medical efficiency and promoting the equalization of public medical services in the pilot areas (24). According to the theory analysis above, the first assumption could be put forward as follows:

**Hypothesis 1 (H1):** The implementation of the hierarchical medical system pilot in Shanghai was able to significantly promote the efficiency of medical resource allocation and performance of medical institutions in pilot districts.

Because there are great developmental differences between central districts and suburban districts in Shanghai, the effects of the hierarchical medical system pilot may have spatial heterogeneity. Medical institutions in central districts are more comprehensive than medical services in suburban districts (24). In addition, local medical departments in suburban districts seem to lack incentives to cooperate in terms of hierarchical treatment measures because of a lack of public supervision (25). According to the current situation, we can also put forward the second hypothesis:

**Hypothesis 2 (H2):** The effects of the hierarchical medical system pilot have regional heterogeneity. Therefore, policy function may be effective in central districts while it is unclear whether this is the case in suburban districts of Shanghai.

To fully measure the effects of the policy pilot in Shanghai, China, it is necessary to identify the dynamic effect of a hierarchical medical system. This project intends to adopt the event study method to carry out empirical analysis. Although the policy pilot shows immediate improvements in the efficiency of medical resource allocation, it remains uncertain whether the effects of a hierarchical medical system would remain. This is because China is still a developing country, and local governments' fiscal resources may not be sufficient to afford hierarchical medical expenditure (26). Furthermore, local governments prefer to increase investment in vanity projects (such as infrastructure construction) instead of medical service investment (27). A hierarchical medical system and medical reform policies that are enforced might not achieve long-term results. As a result of these considerations, a third hypothesis could be suggested as follows:

**Hypothesis 3 (H3):** The hierarchical medical system only had a short-term effect in pilot districts in Shanghai because of huge fiscal expenditure pressure and selective preference of local governments.

This paper intends to explore the influencing mechanisms of the hierarchical medical system to explain its temporal effects. After careful consideration of various aspects, we plan to assess the influencing mechanisms from two dimensions: the expense of the patient and the capacity of medical institutions. Since the sustainability of the policy effects have not been confirmed, it is asserted that these two influencing mechanisms remain uncertain. The last hypothesis could be described as follows:

**Hypothesis 4 (H4):** The influencing mechanisms of reducing the expense of patients and improving the capacity of medical institutions are insufficient to support the enforcement of a hierarchical medical system in Shanghai, and it is suggested that these proposed mechanism variable measures do not produce significantly positive results to promote policy implementation.

**Figure 3:**
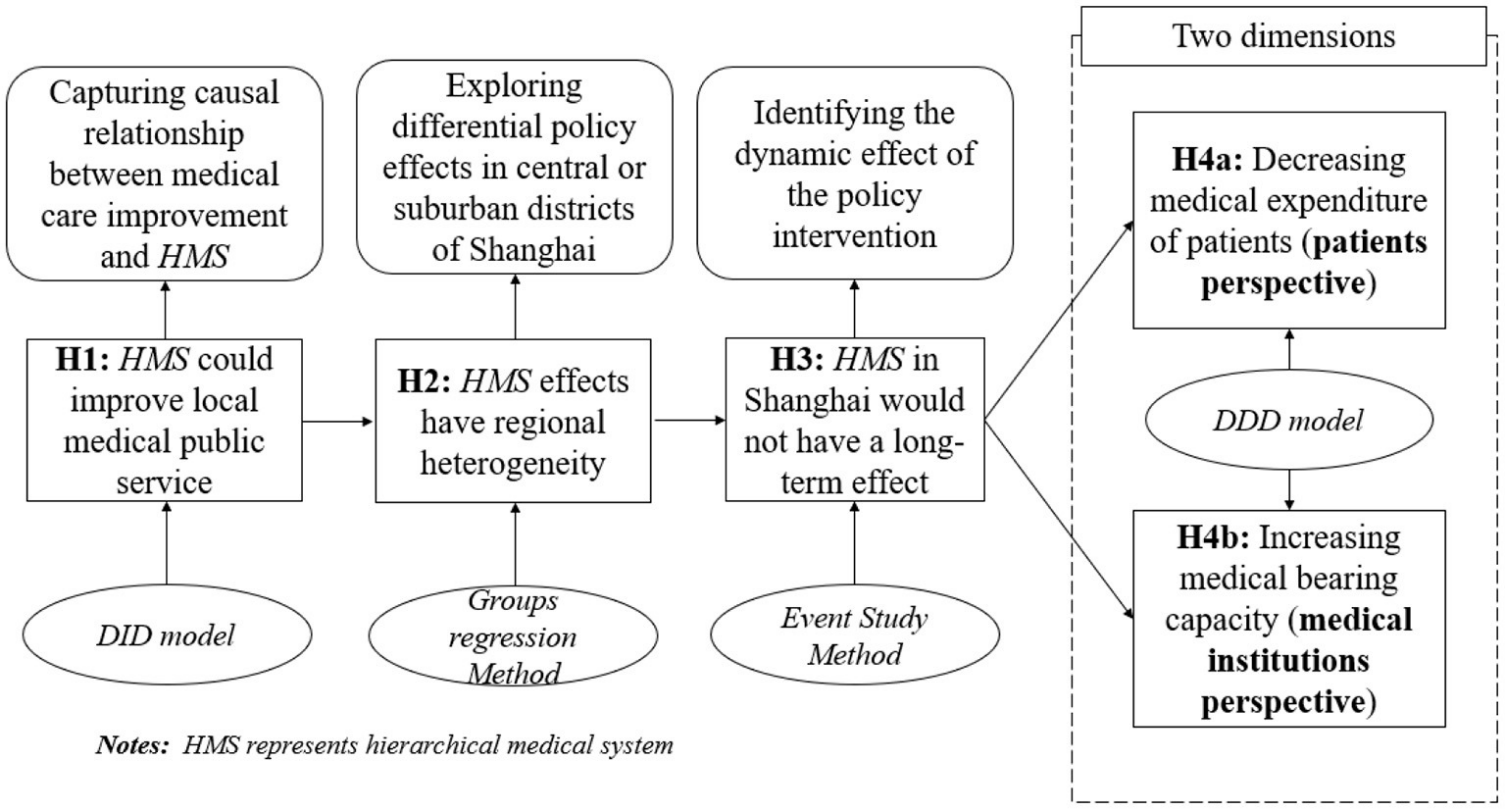
The theoretical framework of this project.

In order to make the logic of this project clearer, the theoretical framework is shown in Figure 3, which includes the research content flow chart, related research methodology, and corresponding explanations.

## Materials and methods

### Sample selection

In 2011, Shanghai implemented the family doctor system, which was regarded as the implementation of hierarchical diagnosis and treatment for the first time. As is shown in Figure 2, there are 17 administrative districts in Shanghai. Ten districts (Changning, Xuhui, Pudong, Jingan, Zhabei (merged with Jingan district in 2015), Baoshan, Yangpu, Minhang, Qingpu, and Jinshan), shown in green, were randomly chosen as the pilot districts, which satisfied the pre-conditions of the DID method. In order to prepare for DID analysis, the pilot districts were assigned as groups receiving treatment, while seven districts, shown in white (non-pilot) were assigned as control groups. The time period for this policy evaluation was from 2005 to 2016, and the number of administrative districts containing treated and control groups is 17. In total, the dataset of this research (panel data) contains 204 (12^*^17) sample data points.

### Methodology

#### Differences-in-differences (DID) and triple differences (DDD)

In this research, since the selection of pilot districts was random, the implementation of a hierarchical medical system was regarded as a natural experiment, which satisfied the pre-conditions of the difference-in-difference (DID) method (28). The common equation of DID is shown in equation (1). The theory of the DID method is that the control groups could generate a possible counterfactual outcome compared to the treated groups, and with the method of comparing the changes between the control and treated groups, we could obtain the net policy effect (29). Thus, the changing nature of dependent variables in pilot districts and non-pilot districts should be constant before the policy intervention (common trend assumption). In order to control other social-economic factors which might affect the empirical result, several control variables were added into the standard model (1) as covariates and the following model could be generated, as seen in Equation (2):


(1) $$\begin{array}{l} Y_{it}=\alpha +\beta Interaction_{it}+\gamma_{1}Treat_{i}+\gamma_{2}{Year2011}_{t}+\epsilon_{it} \end{array}$$

(2) $$\begin{array}{l} Y_{it}=\alpha +\beta Interaction_{it}+\rho \Sigma_{i=2005}^{2016}X_{it}+\mu_{i}+\tau_{t}+\epsilon_{it} \end{array}$$

Where *Y*_*it*_ represents the efficiency of medical resource allocation in district *i* in year *t*, *Interaction*_*it*_ is the dummy variable of the implementation of hierarchical medical system, which can be calculated by $Treat_{i}^{*}{Year2011}_{t}$. *Treat*_*i*_ and *Year*2011_*t*_ are also dummy variables which reflect the district and year features. If a district *i* was attributed into a treated group, *Treat*_*i*_ =1, otherwise *Treat*_*i*_ =0. Meanwhile, if Year *t* was earlier than policy pilot time (2011), *Year*2011_*t*_ =0, otherwise *Year*2011 =1. μ_*i*_ are variables which could indicate city-fixed effects, reflecting the time-invariant characteristics in different districts, such as the natural conditions in Shanghai that can affect policy effects without a change in time. τ_*t*_ are variables that indicate year-fixed effects, capturing the yearly characteristics of all districts in Shanghai, such as the lockdown caused by COVID-19. *X*_*it*_ is the collection of control variables. ϵ_*it*_ is the error term, which is assumed to be normality distributed at zero mean value and constant variance (30, 31). In order to solve the systematic error that stems from serial correlation and unobservable variables, standard errors (S.E.) were clustered at the administrative district level. The most important explanatory variable is *Interaction*_*it*_. The net policy effect could be represented by the coefficient and significance of the core variable.

In order to identify the influencing mechanisms of hierarchical medical systems, this project adopted the triple-differences (DDD) method [equation (3)], which was used to add the dependent variable *mechanism* in equation (2). *Effect* is the dummy variable indicating whether there was an improvement compared to the previous year. Here is the model of the DDD method (32).


(3) $$\begin{array}{l} mechanism=\alpha +\beta Interaction_{it}*Effect+\gamma Interaction_{it}\\ +\rho \Sigma_{i=2012}^{2016}X_{it}+\mu_{i}+\tau_{t}+\epsilon_{it} \end{array}$$

#### Dynamic effect test

In addition, it is necessary to explore the dynamic effect of hierarchical medical systems in Shanghai. Therefore, this project planned to adopt the *event study method* (33, 34) to evaluate the expected and lag effects of this policy implementation. As shown in equation (4), coefficients of city-fixed effects and year-fixed effects were added to control the most unobservable factors in different districts in Shanghai. Dummy variables $Treat_{i}^{*}Year_{t}$ are the interaction terms which reflected the expected and lag effects from 2005 to 2016.


(4) $$Y_{it}\text{​}=\text{​}\alpha \text{​}+\text{​}\Sigma_{j=2012}^{2016}\beta_{j}Treat_{i}\text{​}*\text{​}Year_{t}\text{​}+\text{​}\rho \sum X_{it}+\mu_{i}+\tau_{t}+\epsilon_{it}$$

### Variable selection and data sources

#### Explained variable

This project explored the policy effect from two perspectives. Firstly, the efficiency of medical resource allocation was chosen as one of the dependent variables which could fully reflect the effects of the hierarchical medical system. This project measured the explained variable (*Efficiency*) from the position of supply and demand of medical resources. Indicators *Number of doctors per 10,000 people* and *Number of beds in health institutions per 10,000 people* reflect the supply scale of medical resources, while indicator *Average number of daily patients received by medical institutions* reflects the demand pressure of medical institutions. Secondly, for the accuracy of the empirical results, this project also chose *Average number of daily patients received by medical institutions* as another dependent variable, which was the top-down performance appraisal indictor of the hierarchical medical system as a robustness check, since the most immediate improvement would be to expand the scope and level of medical care.

Dummy variables of the implementation of the hierarchical medical system: *Interaction*_*it*_ is the dummy variable of the implementation of the hierarchical medical system, and it can be calculated by $Treat_{i}^{*}{Year2011}_{t}$. If a district *i* in Shanghai launched the hierarchical medical system in year *t*, in the current time and the future time *Interaction*_*it*_ =1, otherwise, *Interaction*_*it*_ =0.

#### Control variables

This project selected control variables from the parameters of economic variables, social factors, and natural factors (35). (1) From the perspective of economic factors, *per capita GDP, industrial structure* and *government fiscal ability* are used as economic variables. *per capita GDP (per gdp)* reflects the total overall economic level of residents. *Industrial structure* (*ter_gdp*) is calculated as the proportion of added values in GDP of tertiary industries, which may reflect the economic development stage. *Government fiscal ability* (*fis_abil*) is expressed as the proportion of fiscal expenditure in GDP, which reflects the fiscal expenditure level of local governments. (2) From the perspective of social factors, this project selected two control variables: *Population density* and *Proportion of registered population. Population density* (*pop_den*) can be generally measured by total resident population divided by the administrative area; *Proportion of registered population* (*hou_ratio*) is the proportion of the registered population in the resident population. (3) Concerning natural factors, we chose the *green plants' coverage rate (green)* to control the natural conditions.

This project used panel data from 2005 to 2016 in 17 districts in Shanghai (although Zhabei district merged into Jingan district in 2015, *i* will also treat these two regions separately). All of the districts' social and economic data could be found in the *Shanghai Statistical Yearbook*, and the dummy variables could be assigned by referring to the content of the pilot policy. This project also took the continuous variables to their natural logarithm in order to relieve heteroscedasticity. The descriptive statistics of all the explained variables, explanatory variables, and control variables are shown in Table 1.

**Table 1:** Descriptive statistics of all the variables.

**Category**	**Variable**	**Obs**	**Mean**	**Std.Dev**	**Min**	**Max**
DV	efficiency	204	0.354	0.208	0	0.961
DV(RC)	treat_pop	204	55,670.37	30,231.17	14,261.47	139,965.9
	lntreat_pop	204	10.777	0.559	9.565	11.849
KV	Interaction	204	0.294	0.457	0	1
	Treat	204	0.588	0.493	0	1
	Year	204	0.5	0.501	0	1
EV	per_gdp	204	7.936	5.391	1.458	31.439
	ter_gdp	204	0.612	0.242	0.259	0.998
	fis_abi	204	0.109	0.0282	0.0557	0.198
SV	pop_den	204	1.258	1.223	0.0554	4.067
	hou_ratio	204	0.691	0.155	0.354	0.959
NV	green	204	0.305	0.0884	0.122	0.452

DV denotes the dependent variables; DV(RC) denotes the dependent variables in robustness check; KV denotes the key explanatory variables; EV, SV and NV are control variables which represent some variables in economic development, social factors and natural factor respectively.

## Results

### Benchmark results

#### Common trend assumption test

**Figure 4:**
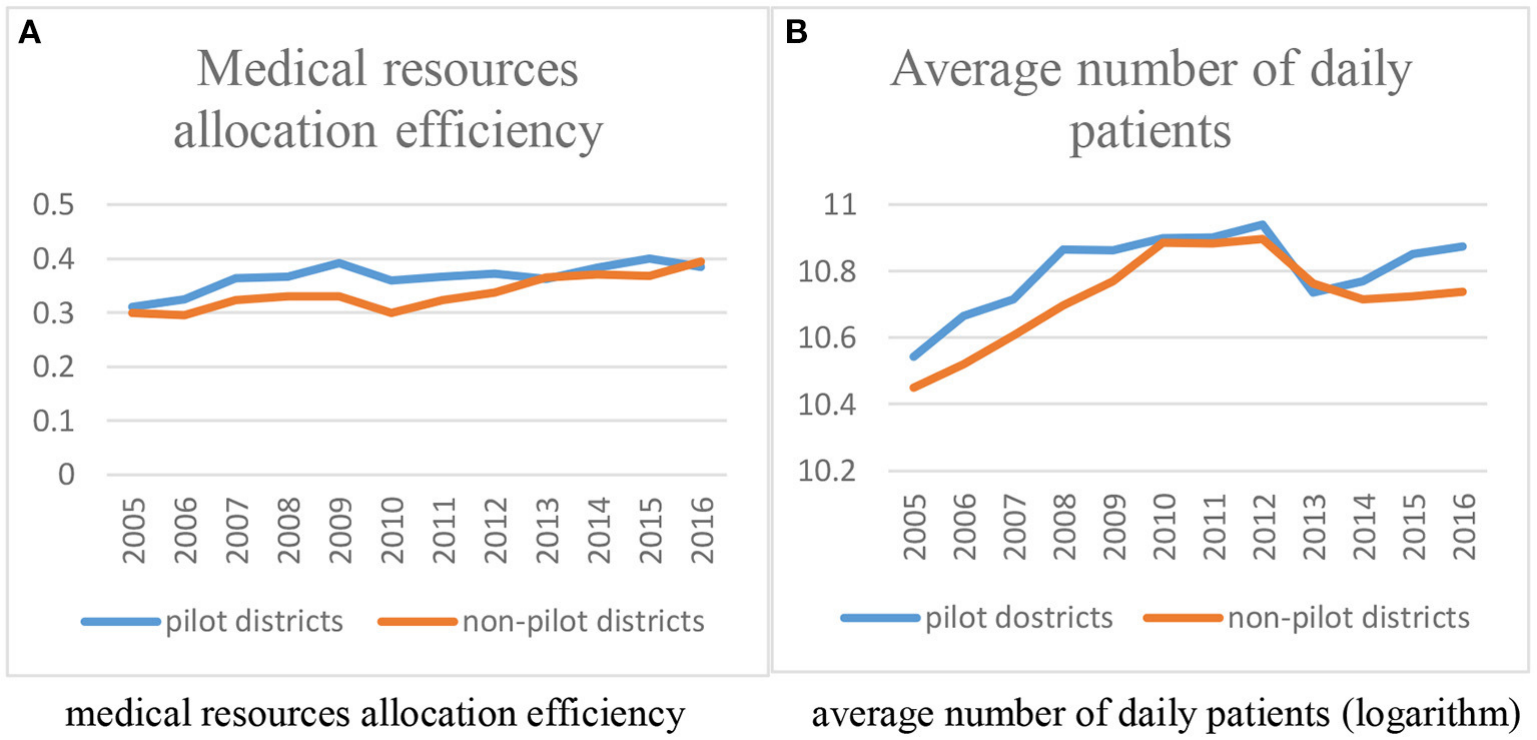
Common trend assumption test of dependent variables before DID regression. **(A)** Medical resource allocation efficiency. **(B)** Average number of daily patients (logarithm).

As seen in Section 4.2 “Methodology”, a precondition of the DID method is that the treated group should have the same changing trend as their counterfactual condition. Within this project, the control group (non-pilot districts in Shanghai) over the same period were taken to replace the counterfactual group. Only if the common trend assumption was satisfied could the DID method provide an effective empirical result. Because the hierarchical medical system in Shanghai was implemented in 2011, this project took 2011 as the starting point from which to identify policy effects. We compared the pre-existing time trend of dependent variables in Figure 4 to confirm whether the common trend assumption is valid or not.

Figure 4 presents the average changing trend of *medical resources allocation efficiency* (4-A) and *average number of daily patients received by medical institutions* (4-B) in the treated group and control group from 2005 to 2016, respectively. As shown in the two figures, these two groups shared same time changing trend before 2011, which meets the common trend assumption. At the same time, in terms of *medical resources allocation efficiency*, the efficiency of non-pilot districts gradually exceeded the efficiency of pilot districts, which indicated that the hierarchical medical system may not improve the allocation of local medical resources. With regard to *average number of daily patients*, it was ascertained that the disparity between the treated group and control group gradually widened after the policy pilot, which may indicate that the hierarchical medical system could improve local hospitals' performance. However, the conclusions that can be drawn based on this information do not reveal a definitive verdict. It is essential that the common trend assumption test (Section Analysis of dynamic effects) is used and that the policy effects are identified (Section Main regression results) with empirical analysis.

#### Main regression results

In order to reduce the deviation coming from unobservable regional and time-dependent variables, this project controlled the district-fixed and year-fixed variables in all of the DID regressions. An evaluation of the policy effects is referred to in Table 2. The dependent variable is *medical resources allocation efficiency*, shown in columns (1) and (2), while dependent variable is *average number of daily patients*, presented in columns (3) and (4).

**Table 2:** Evaluation of the hierarchical medical system policy effects.

	**Efficiency**		**lntreat_pop**	
**Variables**	**(1)**	**(2)**	**(3)**	**(4)**
Interaction	0.0256*	0.0312	0.0837**	0.0509*
	(0.0134)	(0.0219)	(0.0429)	(0.0302)
per_gdp		0.0181***		0.0246***
		(0.00312)		(0.00399)
ter_gdp		0.262		0.402**
		(0.162)		(0.203)
fis_abi		−0.333		−3.096***
		(0.318)		(0.701)
pop_den		−0.0017		0.172***
		(0.0277)		(0.0241)
hou_ratio		0.00001***		0.000005
		(0.000003)		(0.00001
green		−0.0182		−1.658***
		(0.245)		(0.461)
constant	0.346***	0.125	10.752***	10.945***
	(0.0506)	(0.105)	(0.132)	(0.232)
district-fixed effect	Y	Y	Y	Y
year-fixed effect	Y	Y	Y	Y
observations	204	204	204	204
R-squared	0.2215	0.4512	0.1961	0.7653
Number of groups	17	17	17	17

Robust standard errors in parentheses. *p < 0.10, **p < 0.05, ***p < 0.01.

As shown in Table 2, we were able to get the empirical analysis results in columns (1) and (3) from equation (1), which only controls the district-fixed effect and year-fixed effect. The coefficients of *Interaction* (policy effect) are both significantly positive for dependent variables *efficiency* and *lntreat_pop* at 10 and 5%, respectively. Columns (2) and (4) reflect the empirical results controlling for other control variables. The values of R-square both increased, which demonstrated that these control variables are suitable for sharing partial explanatory function from the key explanatory variables when comparing with columns (1) and (3). In terms of *medical resources allocation efficiency*, its estimate coefficient remains positive, but it changes to insignificant. In terms of *average number of daily patients*, its estimate coefficient is robust, and its policy effect is proved to be significantly positive at 10%, which decreases when compared to column (3). According to the main regression results, we can conclude that the hierarchical medical system pilot significantly improved the efficiency of medical resource allocation and the capacity of medical institutions in pilot districts of Shanghai. However, the effect of the policy on medical allocation efficiency might be weaker than its effect on medical institutions' capacity, which indicates that local governments prefer to improve top-down performance indicators. Furthermore, this may gradually lead to campaign-style enforcement is a recognised term in the field of public administration, which could describe the short-term top-down policy effect. In order to fully assess the hierarchical medical system pilot, we will carry out heterogeneity analysis in the following part.

In addition, the regression coefficients before the control variables are entirely as expected. The local per capita GDP correlates significantly positively with dependent variables, which indicated that local residents' life quality clearly rose with improved local medical care. The proportion of tertiary industries' added values in GDP are meanwhile positively correlated with the local medical care service level, since the tertiary industry could also be regarded as another service industry. Government fiscal ability was shown to be significantly negatively correlated with dependent variables; this means that local governments prefer to increase fiscal expenditure in the field of vanity projects such as infrastructure construction, instead of medical public services, when they have more fiscal ability (36, 37). The influence of other unobservable factors, such as the coefficients of population density, proportion of registered population, and green plant coverage rate, on local medical care service levels is unclear.

### Heterogeneity analysis of policy effects

There is a huge gap between central districts and suburban districts regardless of their economic development level, public service allocation, and other social-economic factors. Without doubt, the capacity of medical resources in central districts should be higher than that in suburban districts, and the difference is still widening (38). It is meaningful to carry out heterogeneity analysis to explore the policy function differences of the hierarchical medical system in Shanghai. According to the urban spatial distribution, it is generally accepted that the central districts in Shanghai are Xuhui, Jingan, Changning, Huangpu, Hongkou, Zhabei, Putuo, and Yangpu, while suburban districts are Baoshan, Minhang, Jiading, Songjiang, Qingpu, Jinshan, Pudong, Fengxian, and Chongming. This project divided these districts into two groups (central districts and suburban districts) and carried out group-level regressions to identify the regional differences in the effects of policy. To do this, variable *medical resources allocation efficiency* was chosen as a unique dependent variable to measure the hierarchical medical system pilot effects, since this variable is the most representative variable. The heterogeneity analysis result is shown as follows.

According to the heterogeneity analysis result in Table 3, columns (1) and (2) are regression results of the hierarchical medical system effects in central districts, while columns (3) and (4) are empirical results of the hierarchical medical system effects in suburban districts in Shanghai. Firstly, it can be observed that the regression results are robust, regardless of whether control variables were included or not. Secondly, in terms of the central districts in Shanghai, it can be concluded that the hierarchical medical system pilot improved the efficiency of local medical resource allocation, since their coefficients are all significantly positive at 1%. However, in terms of the policy effects in suburban districts of Shanghai, the coefficients before core variable *Interaction* are lower than the coefficients of central districts. At the same time, these coefficients are all negligibly positive at 10%, which indicated that the policy pilot effect is uncertain in suburban districts of Shanghai. Because the central districts in Shanghai are all advanced in terms of their economic and medical care fields, they have relatively comprehensive medical systems, and therefore it was easier to administer a hierarchical medical system in these areas. Medical institutions in the suburban districts of Shanghai are not at an advanced stage and mainly consist of primary medical institutions and medical departments that do not have the motivation to cooperate in terms of the enforcement of a hierarchical medical system. This project will also fully evaluate the policy pilot from a long-term perspective.

**Table 3:** Heterogeneity analysis of hierarchical medical system pilot effects.

	**Efficiency**			
	**Central districts**		**Suburban districts**	
**Variables**	**(1)**	**(2)**	**(3)**	**(4)**
*Interaction*	0.0751***	0.0472***	0.0116	0.00985
	(0.0223)	(0.0151)	(0.0252)	(0.0276)
*per_gdp*		0.0108***		0.0128
		(0.00235)		(0.00797)
*ter_gdp*		−0.0765		0.179
		(0.191)		(0.195)
*fis_abi*		−0.0515		−0.00383
		(0.297)		(0.480)
*pop_den*		−0.116***		−0.0629
		(0.0329)		(0.156)
*hou_ratio*		−0.181		0.552**
		(0.185)		(0.178)
*green*		0.262		−0.0829
		(0.252)		(0.167)
constant	0.431***	0.803***	0.196***	−0.258
	(0.0187)	(0.261)	(0.0218)	(0.173)
district-fixed effect	Y	Y	Y	Y
year-fixed effect	Y	Y	Y	Y
observations	96	96	108	108
R-squared	0.0451	0.4125	0.0330	0.1724
Number of groups	8	8	9	9

Robust standard errors in parentheses. *p < 0.10, **p < 0.05, ***p < 0.01.

### Analysis of dynamic effects

**Figure 5:**
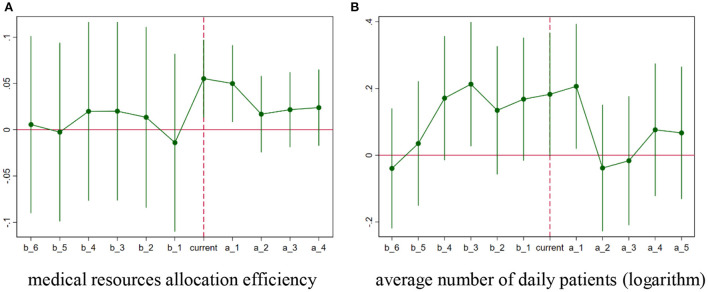
Leading and lagging effects of hierarchical medical system. **(A)** Medical resource allocation efficiency. **(B)** Average number of daily patients (logarithm).

According to the DID analysis results above, it can be concluded that the hierarchical medical system pilot made improvements in the efficiency of local medical resource allocation and standard of medical care. However, it is uncertain whether the hierarchical medical system will have a long-term effect on improving the capacity of local medical institutions. Meanwhile, it is necessary for us to identify whether the policy pilot constitutes campaign-style enforcement. This project added some dummy variables – *b_1, b_2…b_6* and *a_1, a_2…a_5 –* to the key independent variables instead [see equation (4)], which represent the explanatory variables of hierarchical medical system pilot effects in years (one year, two years etc.) before or after the hierarchical medical system implementation, respectively. Variable *current* means the current year of policy implementation. If year *t* is one year before or after the policy implementation, *b_1* or *a_1* =1, otherwise *b_1* or *a_1* =0. If year *t* is two year before or after the policy implementation, *b_2* or *a_2* =1, otherwise *b_2* or *a_2* =0. According to the model above, this project carries out two sets of regressions that control district-fixed and year-fixed effects. The dynamic effect regression analysis results are presented in Table 4, and the changing trends of the policy pilot effects can be seen in Figure 5.

**Table 4:** Dynamic effects of hierarchical medical system.

	**Efficiency**		**lntreat_pop**	
**Variables**	**(1)**	**(2)**	**(3)**	**(4)**
b_6	−0.0389	0.00563	−0.194	−0.0394
	(0.0612)	(0.0583)	(0.276)	(0.109)
b_5	−0.0236	−0.00252	−0.0713	0.0353
	(0.0607)	(0.0588)	(0.276)	(0.113)
b_4	−0.0171	0.0199	0.0772	0.171
	(0.0607)	(0.0588)	(0.276)	(0.113)
b_3	−0.0122	0.0202	0.127	0.213*
	(0.0607)	(0.0588)	(0.275)	(0.113)
b_2	−0.0118	0.0135	0.0751	0.135
	(0.0615)	(0.0595)	(0.276)	(0.117)
b_1	−0.00531	−0.0139	0.131	0.168
	(0.0606)	(0.0585)	(0.275)	(0.112)
current	0.0837**	0.0554**	0.163	0.183
	(0.0369)	(0.0256)	(0.286)	(0.113)
a_1	0.0737**	0.0501**	0.202	0.207*
	(0.0369)	(0.0253)	(0.275)	(0.114)
a_2	0.0291	0.0169	−0.0028	−0.0382
	(0.0367)	(0.0251)	(0.276)	(0.115)
a_3	0.0371	0.0218	0.0319	−0.0164
	(0.0367)	(0.0247)	(0.276)	(0.118)
a_4	0.0479	0.0241	0.114	0.0763
	(0.0371)	(0.0251)	(0.276)	(0.121)
a_5	0.0438	0.0254	0.137	0.067
	(0.0399)	(0.0273)	(0.275)	(0.121)
Control variables	N	Y	N	Y
District-fixed effects	Y	Y	Y	Y
Year-fixed effects	Y	Y	Y	Y
Constant	0.0802	0.101	10.738***	10.495***
	(0.0892)	(0.0794)	(0.205)	(0.245)
N	204	204	204	204
R-squared	0.5062	0.6809	0.1279	0.7757

Robust standard errors in parentheses. *p < 0.10, **p < 0.05, ***p < 0.01.

As shown in Table 4, the two sets of regressions are robust, and the values of R-squared increase when the control variables of equation (4) are added. Thus, we only need to consider columns (2) and (4). Firstly, the regression results confirm the common trend assumption. The coefficients of *b_1* to *b_6* are practically insignificant, which means that there were not significant differences between the treated group and the control group before the implementation of the hierarchical medical system, and the same trend was experienced by each group during the pre-treatment period. Secondly, it is clear that the hierarchical medical system pilot only improved temporarily, regardless of the efficiency of medical resource allocation or the hierarchy level of the medical institution.

Figure 5 further illustrates this conclusion regarding the efficiency of medical resource allocation; the coefficients' changing trend is unstable at the early stage. Immediately following the implementation of the hierarchical medical system pilot, these estimates increase positively, and their 90% confidence interval almost excludes zero in the following 2 years. However, similar to campaign-style enforcement, the estimates become insignificant 2 years after policy implementation. Regarding the hierarchical level of medical institutions, the evolution trend (Figure 5B) showed only small differences compared to Figure 5A; only the coefficient of *a_1* is significantly positive while the 90% confidence interval of other estimates becomes zero. Dynamic effect analysis confirms the short-term effects of the implementation of the hierarchical medical system in Shanghai, which could also be as a result of the method of enforcement.

### Influencing mechanism analysis

According to the empirical results in Section Analysis of dynamic effects, the hierarchical medical system pilot had an immediate effect on improving medical capacity in pilot districts. However, the effects of this policy enforcement will not last long. Local governments carry out campaign-style enforcement which could effectively satisfy their top-down performance appraisal indicators. Although the hierarchical medical system could serve as a policy tool for local officials to improve public medical services, in the actual policy implementation stage, neither the policy process nor the policy result can produce a legal effect, since the hierarchical medical system is only an administrative measure that has no mandatory adherence requirement. As mentioned above, the pilot effects of the hierarchical medical system might gradually decline after policy implementation.

In addition to identifying the temporary effects of the hierarchical medical system, it is necessary for us to explore the inner mechanisms of the hierarchical medical system that may influence why the pilot only had a temporary effect. This project will assess the influencing mechanisms from two dimensions. Considering patients that received treatment, *Per capita medical care expenditure* was used to measure the medical expenses of every patient. We also consider its natural logarithm to weaken heteroscedasticity. Considering the medical institutions, *medical capacity* was calculated to measure the efforts the medical institutions made. It contains the indicators *Number of doctors per 10,000 people, Number of beds in health institutions per 10,000 people*, and *number of medical institutions*; the entropy method was adopted to standardize the indicators above. All of the data and factors were collected from the *Shanghai Statistical Yearbook* from 2006 to 2017. Two sets of regressions were run, which control the district-fixed and year-fixed effects, and the empirical results can be found in Table 5.

**Table 5:** Influencing mechanism analysis of hierarchical medical system.

	**lnper_expe**		**Capacity**	
**Variables**	**(1)**	**(2)**	**(3)**	**(4)**
Interaction	0.0701	0.0778	−0.0329*	−0.0453***
	(0.0754)	(0.0727)	(0.0172)	(0.0141)
per_gdp		0.0411***		0.0139***
		(0.0112)		(0.0022)
ter_gdp		−0.0416		0.185
		(0.610)		(0.115)
fis_abi		0.309		0.815***
		(1.343)		(0.115)
pop_den		−0.149		−0.0565
		(0.185)		(−0.035)
hou_ratio		0.00004		0.226**
		(0.00006)		(0.0929)
green		0.919		0.352***
		(0.605)		(0.115)
constant	5.653***	5.141***	0.241***	−0.238**
	(0.101)	(0.333)	(0.0146)	(0.104)
District-fixed effect	Y	Y	Y	Y
Year-fixed effect	Y	Y	Y	Y
observations	204	204	204	204
R-squared	0.6155	0.8425	0.5831	0.7609
number of groups	17	17	17	17

Robust standard errors in parentheses. *p < 0.10, **p < 0.05, ***p < 0.01.

As shown in Table 5, the regression results are robust when adding these control variables. Considering that the values of R-square increase after adding control variables to carry out regression, we only need to consider the results in columns (2) and (4). In terms of *Per capita medical care expenditure*, its regression coefficients are insignificantly positive: there is no direct evidence indicating that the per capita medical expenditure decreased after the implementation of the hierarchical medical system. At the same time, the coefficients of *medical capacity* are negative and significant, which means that the hierarchical medical system not only did not improve the medical care capacity in pilot districts but also significantly weakened the allocation of local medical resources. This is an urgent situation that local medical departments need to consider. From the above analysis of influencing mechanisms, we can explain why the hierarchical medical system only had short-term policy effects. When a top-down administrative order is required, local governments might use campaign-style enforcement to achieve political goals, with dominant power and abundant administrative resources (39). The consistent enforcement of policy will decline as time goes on. In the hierarchical medical system pilot, although continuing for 2 years after implementation, the policy effect did not last long because of governance approaches such as unreasonable resource allocation and maintaining treatment expenditure of patients.

## Discussion

In order to accurately calculate the effects of the hierarchical medical system policy pilot in Shanghai, this project first used the DID method to evaluate the average treatment effects (ATE). According to the DID regressions results showing in Table 2, it can be concluded that the hierarchical medical system in Shanghai significantly improved local medical care service levels, regardless of *medical resources allocation efficiency* and *average number of daily patients* being dependent variables, which is consistent with previously published research (40, 41). The hierarchical medical system in Shanghai generally promoted the process of medical institutions conducting two-way referrals and treatment by primary institutions. However, the policy effect on the efficiency of medical resource allocation (core indicator) is weaker than its effect on the hierarchy level of the medical institution (top-down assessment indicator), which indicates that local governments prefer to improve top-down performance indicators (42). According to Chinese campaign-enforcement theorists, this might gradually develop into campaign-style medical reform. Therefore, it is necessary to adopt a strategy to verify and explore the long-term effects.

Secondly, by means of heterogeneity analysis (the project only selected the efficiency of medical resource allocation as a unique dependent variable), it was established that the hierarchical medical system significantly increased the efficiency of medical resource allocation in central districts, while the effect of the pilot in suburban districts was unclear. It was demonstrated that medical institutions in suburban districts have less motivation to cooperate with hierarchical treatment enforcement compared to medical departments in central districts.

Thirdly, in terms of dynamic effects analysis, the event study method was also used to identify the long-term effects of the hierarchical medical system pilot. According to the regression results in Table 4 and the time-changing trend in Figure 5, although these estimate coefficients are significantly positive in the first 2 months after the hierarchical medical system pilot, similar to campaign-style enforcement, the estimates become insignificant 2 years after policy implementation, which could provide empirical evidence for the temporary policy effects of the hierarchical medical system pilot. It is necessary to take immediate action to avoid campaign-style medical reform in China (41).

Finally, it would be worthwhile to clarify what the influencing mechanisms of the hierarchical medical system pilot in Shanghai are, which may explain the temporary effects of the policy pilot and calculate its potential correlations. Table 5 reports the regression results from two perspectives, including *Per capita medical care expenditure* (treatment patient dimension) and *medical capacity* (medical institution dimension). Concerning the policy effects on treatment patients, it can be concluded that there is no direct evidence to prove that the hierarchical medical system pilot could decrease medical expenditure of local patients. Concerning the policy's effects on medical institutions, the estimated coefficients are significantly negative, which indicates that the hierarchical medical system pilot not only could not improve local medical care service capacity but also totally depleted the resources of local medical institutions. These conclusions could explain the dynamic effect analysis results above. Although the hierarchical medical system pilot had an effect in the 2 years after implementation, the policy effects should be observed over a longer period of time because of governance approaches such as unreasonable resource allocation and requiring expenditure of patients for treatment (43, 44).

## Conclusion

Through analysis of the empirical results, we can conclude the following. First, in order to confirm the effects of the policy, the hierarchical medical system should continue to be advanced in terms of system development and implementation to improve the efficiency of local medical resource allocation and medical security capabilities. Secondly, the huge gap between public medical services in central districts and suburban districts in Shanghai cannot be ignored. This would lead to hierarchical medical policy affecting heterogeneity. Suburban districts should be equipped with all ranks of medical institution and promote the equalization of medical public services. Thirdly, with regard to dynamic policy effects of the pilot, the hierarchical medical system with campaign-style enforcement only had short-term effects on improvements. A long-term supervision mechanism should be a priority in the enforcement process of the hierarchical medical system. In addition, because of the fiscal investment preference of local governments, medical care services are usually ignored compared with other infrastructure investments, and it is crucial that appraisal and reform of top-down local government performance in China is carried out; medical service quality should be adequately provided for in the government's performance evaluation system. Finally, based on analysis of the influencing mechanisms, the short-term policy effects were explained from the perspectives of patients and medical institutions. A hierarchical medical system should promote the reduction of patients' medical expenditure and strengthen the capacity of local medical institutions. More safeguarding measures should be implemented, such as supervising the fee structure of medical institutions and conducting performance evaluation.

There may be limitations in the timeliness of our data. The time period of this study ranged from 2005 to 2016 and therefore does not include the latest statistical yearbook. However, the policy pilot was implemented in 2011, at the mid-point of the study period, and the results should therefore be accurate. Future research and evaluation of the hierarchical medical system based on nationwide data is planned.

## Data availability statement

The original contributions presented in the study are included in the article/supplementary material, further inquiries can be directed to the corresponding author.

## Author contributions

CL and YZ conceived and designed the study and revised the paper. CL, CY, and LY analyzed the data. CL and PS wrote the paper. All the authors read and approved the final manuscript.

## Funding

This paper was funded by the National Social Science Foundation of China (19BZZ045).

## Conflict of interest

The authors declare that the research was conducted in the absence of any commercial or financial relationships that could be construed as a potential conflict of interest.

## Publisher's note

All claims expressed in this article are solely those of the authors and do not necessarily represent those of their affiliated organizations, or those of the publisher, the editors and the reviewers. Any product that may be evaluated in this article, or claim that may be made by its manufacturer, is not guaranteed or endorsed by the publisher.

## Footnotes

^1^Referring to 2019 Shanghai Statistical Yearbook: http://tjj.sh.gov.cn/tjnj/20200427/4aa08fba106d45fda6cb39817d961c98.html.

^2^Referring to 2015 Shanghai Statistical Yearbook: http://tjj.sh.gov.cn/tjnj/20170629/0014-1000201.html.

^3^Referring to: Hospital Statistics 1996 1997, edition. American Hospital Association. Chicago. 1997.

^4^Referring to: http://www.bundesaerztekammer.de/specialdownloads/Stat10Abbildungsteil.pdf.

^5^Related information could refer to: http://www.hbyxjzcg.cn/policy/show416.html.
